# Phenotypic, Functional, and Safety Control at Preimplantation Phase of MSC-Based Therapy

**DOI:** 10.1155/2016/2514917

**Published:** 2016-08-29

**Authors:** Wioletta Lech, Anna Figiel-Dabrowska, Anna Sarnowska, Katarzyna Drela, Patrycja Obtulowicz, Bartlomiej Henryk Noszczyk, Leonora Buzanska, Krystyna Domanska-Janik

**Affiliations:** ^1^Mossakowski Medical Research Centre, Polish Academy of Sciences, 5 Pawinskiego Street, 02-106 Warsaw, Poland; ^2^Department of Plastic Surgery at the Medical Center for Postgraduate Education, Warsaw School of Medicine, 01-828 Warsaw, Poland

## Abstract

Mesenchymal stem cells (MSC) exhibit enormous heterogeneity which can modify their regenerative properties and therefore influence therapeutic effectiveness as well as safety of these cells transplantation. In addition the high phenotypic plasticity of MSC population makes it enormously sensitive to any changes in environmental properties including fluctuation in oxygen concentration. We have shown here that lowering oxygen level far below air atmosphere has a beneficial impact on various parameters characteristic for umbilical cord Wharton Jelly- (WJ-) MSC and adipose tissue- (AD-) derived MSC cultures. This includes their cellular composition, rate of proliferation, and maintenance of stemness properties together with commitment to cell differentiation toward mesodermal and neural lineages. In addition, the culture genomic stability increased significantly during long-term cell passaging and eventually protected cells against spontaneous transformation. Also by comparing of two routinely used methods of MSCs isolation (mechanical versus enzymatic) we have found substantial divergence arising between cell culture properties increasing along the time of cultivation* in vitro*. Thus, in this paper we highlight the urgent necessity to develop the more sensitive and selective methods for prediction and control cells fate and functioning during the time of growth* in vitro*.

## 1. Introduction 

MSC cultures exhibit enormous heterogeneity which can influence their therapeutic efficacy as well as safety after transplantation. Difficulty in prediction of biochemical and functional diversity depends not only on differences in individual cell donors, their age, and health [1–3], but also on the tissue of origin and still poorly controlled variations in the methods of cell derivation, time of culture, and discrete environmental changes additionally employed to* in vitro* procedures.

Nowadays, since we approached era of increasing application of MSC therapy in a clinic, standardization of cell manufacturing with optimized isolation efficacy, rate of cell proliferation, and viability and longevity of culture with capacity to differentiation into desired lineages seems to be of key practical issues. All these important demands are not adequately considered and verified by routinely used standard MSC culture preimplantation screening still based on the ISSCR criteria established by International Society for Stem Cell Research in 2008. On the basis of our recent work on establishment of optimized protocols for efficient isolation and cultivation of MSC for clinical purposes we have found that the above criteria failed to detect subtle culture defects which upon time of culture can dangerously accumulate and finally exclude these cells from therapeutic use. Comparing two standard methods frequently employed for MSC isolation we have found substantial divergences between the cell features appearing along the time. Thus, in this paper we highlight the urgent necessity for more sensitive and selective methods for controlling or even better for predicting the cell fate dangerous changes during passaging. At the end we will propose the simple and easy method to prevent appearance of the most adverse events faced during long cell culture like failure of proliferation or cell genome transformation. This can be achieved, as we present it for AD-MSC culture experiments, simply by changing oxygen environment from 21% to ≤5% O_2_ concentration which physiologically occurs in the majority of tissues and stem cell niches* in vivo* [4].

## 2. Material and Methods 

### 2.1. Mechanical Method of WJ-MSC Isolation

Human umbilical cords (15–20 cm long) were acquired from full-term deliveries with the consent of mother according to the Ethics Committee of Warsaw Medical University guideline (KB 33/2012). Cords were washed thoroughly in PBS (*Phosphate-Buffered Saline*; Gibco) with Penicillin-Streptomycin (1 : 100, Gibco) and cut into 2-3 mm slices with sterile scalpel. Avoiding the blood vessels, the cylindrical fragments were cut from the pieces of umbilical stroma using the 3 mm diameter biopsy punch (Miltex, GmbH). Wharton's Jelly fragments were moved into the 25 cm^2^ culture flasks. Scraps obtained from 5 slices of cord were transferred into the one flask in 2 mL growth medium (MSCGM Bullet Kit, Lonza) per dish and cultured for 14 days in a humidified incubator under 21% O_2_ and 5% CO_2_ or 5% O_2_ and 5% CO_2_ atmosphere at 37°C.

The medium was replaced every 3 days until 14th day of culture when the cells coming out from explants were trypsinized by 0.05% Trypsin-EDTA (Gibco) and counted.

### 2.2. Enzymatic Method of WJ-MSC Isolation

The umbilical cord was cut into fragments as above then additionally crushed into smaller parts, carefully skipping the umbilical vessels and perivascular regions. The chopped tissue was placed into 15 mL tubes where each of the tube contained fragments from the 5 slices of the umbilical cord. For enzymatic digestion collagenase NB 4 Standard Grade solution (Serva) at a final concentration of 0.3 U/PBS 7 mL was used followed by trypsinization with (0.05% Trypsin-EDTA) in PBS (50 : 50). The tissue placed into the tubes firstly was incubated 3 hours in collagenase solution thoroughly shaken on an orbital shaker at constant low speed 75 rpm at 37°C. Then the tissue was washed twice with PBS and centrifuged for 5 minutes at 250 ×g. The fragments of cord were poured by Trypsin solution and shaken at 37°C for 30 minutes according to method described by Rodríguez-Fuentes et al. [5] slightly modified in our lab. The final samples were washed again with PBS and transferred immediately into the culture dishes in the MSC growth medium (Lonza). After 48 hours the adhered, fibroblast-shaped cells were separated from the rest of floating debris, rinsed, and cultured for the next 14 days like the previously described mechanically isolated cells.

### 2.3. Adipose Derived Mesenchymal Stromal Cells (AD-MSC) Isolation

AD-MSC were isolated with CellCelution System (Cytori Therapeutics Inc., San Diego, CA). Adipose tissue was collected after a manual liposuction procedure. The subcutaneous fat from abdominal part was harvested in the operating theatre following infiltration with Ringer lactate, Lignocaine, and Adrenaline. Lipoaspirate was immediately transferred into the CellCelution 800 system, then washed again to remove free blood and lipid, and digested with the manufacturer enzyme preparation, the Celase 800 (Cytori Therapeutics, Inc.) to release the stromal vascular fraction. After series of programed centrifugations and passing through a system of sieves with different pores stromal cell fraction was concentrated to 5 mL in Ringer solution. Cells were counted and transferred into the flasks in 2 mL growth medium (MSCGM Bullet Kit, Lonza) and cultured in a humidified incubator under either 5% or 21% O_2_ with 5% CO_2_ in atmosphere at 37°C. The growth medium was replaced every 3 days and the cells were trypsinized when culture confluence reached 70%.

### 2.4. Flow Cytometry Analysis

To detach cells from the culture dishes, Accutase Cell Detachment Solution (Becton Dickinson) was used. Centrifuged cells (1 × 10^6^) required for the analysis were suspended in cold Stain Buffer (Becton Dickinson).

Using Human MSC Analysis Kit (Becton Dickinson), according to manufacturer's protocol, to each tube was added the appropriate dilution of fluorochrome-conjugated antibodies directed against APC CD73, FITC CD90, and PerCP-Cy*™*5.5 CD105 (positive markers) and PE CD34, PE CD11b, PE CD19, PE CD45, and PE HLA-DR (negative markers) and incubated for 30 minutes at room temperature, protected from light.

After incubation, cells were washed twice with Stain Buffer, suspended in 500 *μ*L of this buffer and immediately analyzed using FACSCalibur II cytometer (Becton Dickinson). 10.000 events were counted using FACSDiva software computer program.

### 2.5. Mesodermal and Neural Lineage Differentiation

To evaluate cells capability of mesodermal lineage differentiation, WJ-MSC or AD-MSC at 2nd or 3rd passage were seeded in 24-well plates at suitable density (adipogenesis: 1 × 10^4^ cells/cm^2^, osteogenesis: 5 × 10^3^ cells/cm^2^, and chondrogenesis: 1.6 × 10^7^ cells/cm^2^) and cultured in MSC growth medium (Lonza) under 21% O_2_ until 70% of cell confluence.

For evaluation of the spontaneous WJ-MSC potential for differentiation toward neural lineage the cells were cultured under air atmosphere in condition described in the previous paper [6].

### 2.6. Adipogenesis

When cells reached the proper confluence, the medium was replaced with differentiation medium from Adipogenesis Differentiation Kit (Gibco). After 14 days of differentiation, cells were fixed with 4% PFA for 30 minutes and washed with PBS and then 60% isopropanol was added for 5 minutes.

Staining was made of 99% isopropanol and Oil Red O (Sigma-Aldrich). The resulting solution was diluted in distilled water (3 : 2). Isopropanol was removed and the staining was added after 10 minutes of incubation on the cells for 5 minutes to verify positive effect of differentiation.

### 2.7. Osteogenesis

To induce differentiation into osteogenic lineage, the Osteogenesis Differentiation Kit (Gibco) was used. After 21–25 days of culture in specific medium replaced every 3 days, WJ-MSC and AD-MSC were fixed with 4% PFA for 30 minutes.

Fixed cells were washed twice with distilled water and then stained with 2% Alizarin Red S (Sigma-Aldrich). For staining of the cultures, prepared dye solution was applied for 2-3 minutes and cells were rinsed with distilled water at the end.

### 2.8. Chondrogenesis

Cartilage differentiation was induced by Chondrogenesis Differentiation Medium (Gibco). Micromass pellet of cells was generating in the center of well plate. After 1 hour of incubation in 37°C the Differentiation Medium was added and culture was continued for additional 14 days with medium replaced every 2-3 days. Subsequently cells were fixed with 4% PFA for 30 minutes and washed with PBS.

To demonstrate positive effect of differentiation into chondrocytes cells were stained with 1% Alcian Blue solution (Sigma-Aldrich). Fixed WJ-MSC or AD-MSC were stained with prepared solution for 30 minutes and washed three times with 0.1 N HCl. At the end, to neutralize acidity the cells were rinsed with distilled water.

### 2.9. Immunocytochemistry

For immunocytochemical analysis cells were seeded on 24-well plates covered with poly-L-lysine (Sigma-Aldrich) at density 5 × 10^3^ cells/cm^2^. After approximately 70% confluence, the growth medium was removed, cells were rinsed with PBS, and then the cultures were fixed with 4% PFA for 15 minutes and again washed with PBS. To label intracellular marker proteins 0.2% of Triton X-100 (Sigma-Aldrich) solution was added for 15 minutes. Subsequently nonspecific antigen epitopes were blocked with mixture of 10% Goat Serum (Sigma-Aldrich) and 1% Bovine Serum Albumin (Sigma-Aldrich) by one hour and rinsed with PBS then primary antibodies (Table 1) were applied for 24 hours at 4°C. The following day, cultures were washed thoroughly with PBS and the secondary antibodies (Alexa Fluor 488 for green staining or Alexa Fluor 546 for red staining; in dilution 1 : 750, Invitrogen) were added for one hour. Cells were rinsed with PBS and cell nuclei were stained with Hoechst 33258 dye (1 : 150, Sigma-Aldrich) for 15 minutes.

Immunolabeled cultures were analyzed with Axio Vert.A1 (Zeiss) fluorescent microscope with AxioCam MRc5 (Zeiss) digital camera.

### 2.10. Cell Proliferation Analysis

The routine proliferation analysis was performed by Population Doubling Time (PDT) as described previously [6] starting either from the first passages for WJ-MSC growth rate estimation or after third passages in the case of AD-MSC measurements. Shortly, PDT was calculated based on the total cell number at each passage using the formula (*t* − *t*_0_) × log⁡2/(log⁡*N* − log⁡*N*_0_). The duration of passage counted in days was equal to *t* − *t*_0_. The *N* was the number of cells obtained at the end of particular passage whereas *N*_0_ was the number of seeded cells.

The growth of living cells was estimated by analysis of every day changes in sample metabolic activity with WST-1 reagent (Roche). WJ-MSC at 5th passage obtained with mechanical or enzymatic method were seeded on 96-well plates at density 1500 cells/cm^2^ and every day of one week the enzyme activity was estimated in 6 parallel samples. To each sample 10 *μ*L of WST-1 solution was added and incubated avoiding light, in an incubator at 37°C for 2 hours. The changes in the reduction of tetrazolium salt to soluble formazan by mitochondrial dehydrogenases, being proportional to the number of the viable cells, were analyzed spectrophotometrically at 420 nm using Omega Plate Reader (BMG LABTECH). The curve of cell growth was plotted over the time at *x*-axis and cell number at *y*-axis. All experiments were repeated three times in duplicate.

### 2.11. Senescence Assay

Cells were analyzed using Senescence Cells Histochemical Staining Kit (Sigma-Aldrich). Cultured cells were washed twice with 1x PBS and fixed with fixation buffer for 6-7 minutes at room temperature. After fixation cells were washed 3 times with 1x PBS and the Staining Solution prepared according to manufacturer's protocol was added. The cells were incubated overnight at 37°C. Total number and blue-stained cells were counted and percentage of Stem Associated *β*-Galactosidase (SA-*β*gal) positive cells was calculated.

### 2.12. CFU Assay

The cultured cells were seeded on 6-well plates at density 50 cells/cm^2^. After 14 days cells were washed with PBS, fixed with 4% PFA for 15 minutes, and again washed carefully with PBS. Subsequently cells were stained with 1% toluidine blue prepared in 70% ethanol for 30 minutes and rinsed once with distilled water and the number of stained colonies was counted. CFU frequency was calculated as percentage of seeded cells.

### 2.13. Quantitative RT-PCR Analysis

In the first step mRNA from minimum 1 × 10^5^ cells was isolated using TRIzol Reagent (Invitrogen). Prepared material was resuspended in 30 *μ*L DEPC water (Ambion) and its purity was measured using spectrophotometer NanoDrop ND-1000. Every sample was treated with specific 1 U/mL DNase using DNA-*free* Kit (Ambion) and then reverse transcription reaction was made using the High Capacity RNA-to-cDNA Kit (Applied Biosystems) according to manufacturer's protocol.

Quantitative RT-PCR was analyzed using thermocycler 7500 Real Time PCR System (Applied Biosystems) with cDNA samples, SYBR Green PCR Master Mix (Life Technologies), and specific primers shown in Table 2. For the final results, the expression was calculated by 2^−ΔΔCt^ method with *β*-actin (ACTB) used as an internal control.

### 2.14. Chromosome Analysis

The AD-MSC cultures at the late passages were treated with 5.5 *μ*g/mL Colcemid for 20 minutes in 37°C to stop cell divisions. The next step was hypotonization in 0.075 M solution of KCl (MERCK, 1.04936.1000) for 20–40 minutes, in 37°C. Then cells were fixated with Carnoy's mixture precooled to −20°C (methanol (MERCK, 106009.2511) with acetic acid (SIGMA, A6283) in proportions 3 : 1). Fixated cells were scraped and in small volume of fixation buffer (20 *μ*L) delocalized on slides. The slides were stained according to modified Wang and Fedoroff's method (GTG: G-bands by Trypsin using Giemsa-stain) for bands visualization. This method embraced digestion with 0.1 M HCl (SIGMA, 115752837) for 2-3 seconds, incubation in 2x SSC solution (Standard Sodium Citrate, SIGMA, S4641) at pH 7, for 15 minutes, in 60°C, and staining with 0.25% Wrights solution (SIGMA, 861375) dissolved in phosphate buffer (SIGMA) at pH 6.8, in proportions 1 : 3 for 2-3 minutes. After every group of steps of staining, slides were washed under tap water and dried by warm airflow.

The stained slides were digitalized then observed metaphases were photographed and analyzed in 250x enlargement by IKAROS software of Meta Systems, system for picture analysis.

Karyotyping was done according to International System for Human Cytogenetic Nomenclature's guidelines (ISCN, 2013).

### 2.15. Statistics

Statistical analysis of the raw data was conducted using GraphPad Prism 5 software. The mean ± SD was calculated for all samples and significance was determined using Student's* t*-test. The values were considered significant with *p* < 0.05.

## 3. Results

### 3.1. Effect of Two Different Isolation Methods on the Final Features of Umbilical Cord Culture

#### 3.1.1. Isolation Efficiency

**Figure 1:**
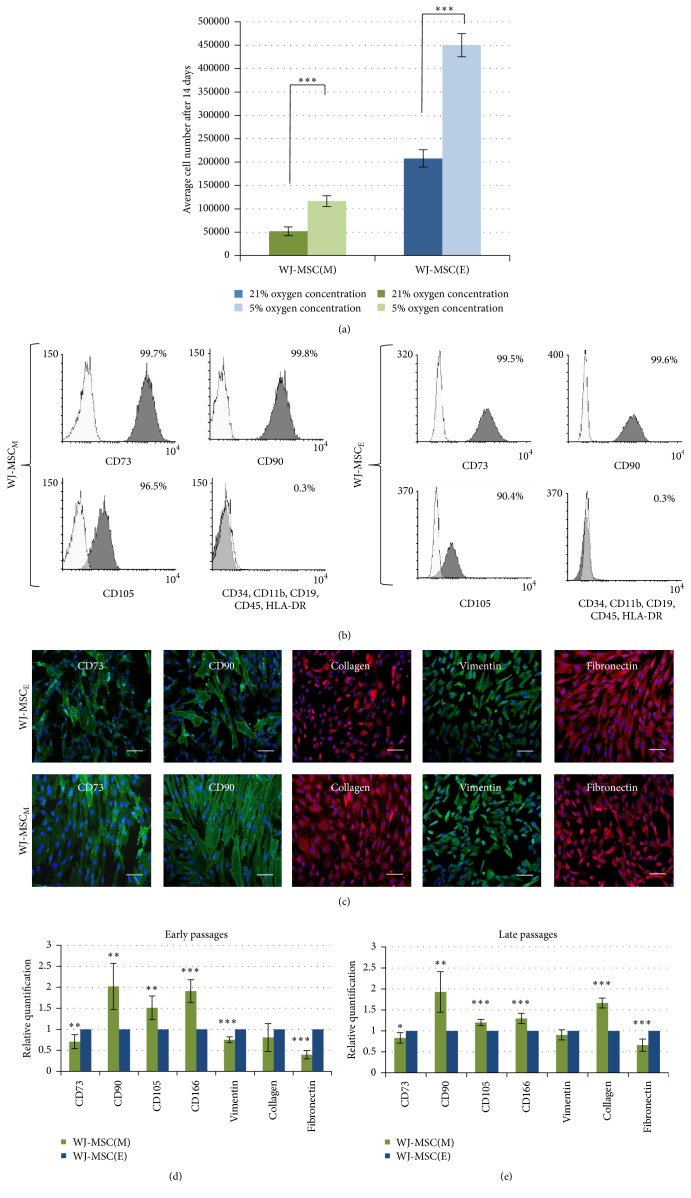
(a) Isolation efficiency. Average number of cells isolated by enzymatic method WJ-MSC(E) is initially higher than cells obtained by mechanical method WJ-MSC(M). Furthermore, under 5% O_2_, the number of received cells significantly increases in all variants of isolation. The results are presented as mean values of 3 isolations ± SD, ^*∗∗∗*^ *p* < 0.001. (b) Flow cytometry analysis. Flow cytometry analysis showed in both types of cultures almost identical typical and relatively high expression of specific mesenchymal markers (CD73, CD90, and CD105). Similarly, not more than 1% of WJ-MSC(E) express negative markers (CD34, CD11b, CD19, CD45, and HLA-DR). (c) Immunocytochemical analysis of WJ-MSC(M) and WJ-MSC(E). Expression of the classical mesenchymal markers revealed presence of the discrete but detectable differences for both types of culture at early passages. (d, e) Quantitative analysis of mesenchymal marker genes expression in early/late passages. Relative expression of WJ-MSC(M) at early (2nd-3rd) and late (8th) passages normalized to the reference gene ACTB was compared to WJ-MSC(E) as calibrator group. The results are presented as mean values of 3 isolations ± SD; ^*∗*^ *p* < 0.05; ^*∗∗*^ *p* < 0.01; ^*∗∗∗*^ *p* < 0.001.

WJ-MSC were isolated by either mechanical- (M-) or enzymatic- (E-) based procedures as described in Section 2 and then cultured for 14 days under 21% O_2_ (an air atmosphere) or 5% O_2_ (an average* in vivo* physioxic environment). This initial cell growth is presented in Figure 1(a) as average cell number attained during this first, 14-day long primary cell culture.

At the beginning of our observation enzymatic (WJ-MSC_E_) isolation seemed to be much more efficient, outnumbering about 4 times the results of mechanical procedure. Proportional increase of cell proliferation rate under physioxia versus classical air atmosphere [6] was similar for both isolates. Furthermore, the flow cytometry analysis (according to ISCT recommendations) showed typical and almost identical mesenchymal characteristic in both types of cultures which did not differ substantially at these early time points up to 2nd-3rd passage (Figure 1(b)).

Considering the data from three first passages it may be concluded that enzymatic method (WJ-MSC_E_) should be rather recommend for further standardization and implementation of cell derivation procedure. However, the further experiments evidently changed our opinion about therapeutic usefulness of both types of explored cultures. After 3rd passage we have observed significant acceleration of the growth rate of mechanically isolated WJ-MSC_M_ in comparison with decreasing dynamics in WJ-MSC_E_ culture. In parallel, comparative analysis of immunocytochemically labeled cells revealed presence of the discrete but detectable differences in the intensity of certain markers expression typical for MSC at this step for both types of culture at early passages (Figure 1(c)). Thus, we have estimated the relevant marker gene expression in early (the second) and late (the eight) passages (Figures 1(d) and 1(e)). Results from early passages confirmed previous immunocytochemical analysis. In both time points we have noticed significantly higher expression of the MSC-specific markers CD90, CD105, and CD166 in mechanically isolated WJ-MSC_M_, while expression of CD73, vimentin, and fibronectin was found slightly lowered there. This tendency did not change significantly in the 8th (late) passage, except for increase of collagen mRNA expression in this WJ-MSC_M_ passage (Figures 1(d) and 1(e)).

#### 3.1.2. Cell Proliferation and Senescence Analysis

**Figure 2:**
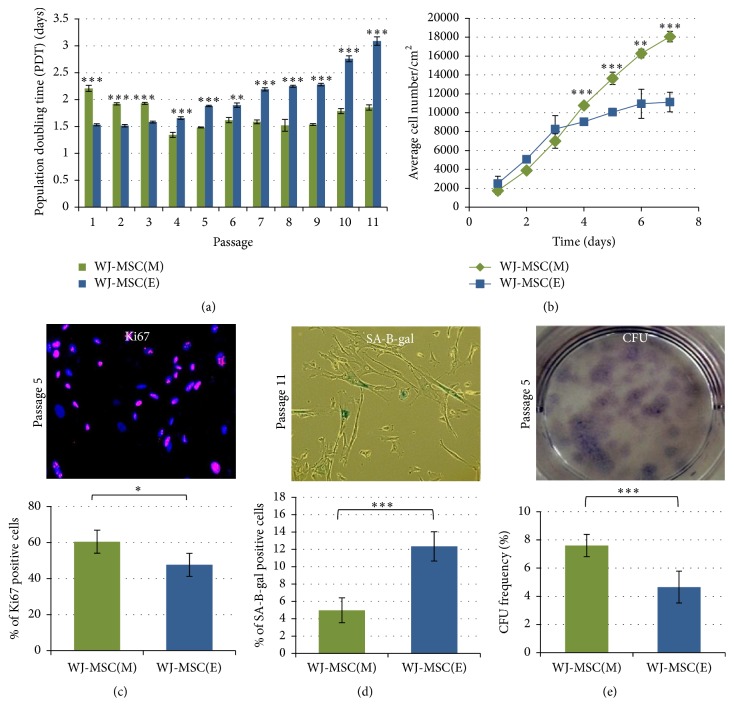
(a) Comparison of WJ-MSC(M) and WJ-MSC(E) population doubling time. Up to 11th passage PDT in WJ-MSC(M) remained at a relatively stable level of 1.5–2 days. In contrast, the WJ-MSC(E) have slowed down their cell divisions at the latest 3 passages (9–11). The results are mean values of 3 experiments ± SD. ^*∗∗*^ *p* < 0.01; ^*∗∗∗*^ *p* < 0.001. (b) Cell growth analysis. Only WJ-MSC(M) presents a linear growth rate. After 7 days of culture the average number of WJ-MSC(E) was significantly lower than that of WJ-MSC(M). The results are mean values of 3 experiments ± SD; ^*∗∗*^ *p* < 0.01; ^*∗∗∗*^ *p* < 0.001. (c) Cell proliferation. Decrease of WJ-MSC(E) proliferation rate correlated with decreased number of Ki67 reactivity. The results are mean values of 3 experiments ± SD. ^*∗*^ *p* < 0.05 (d) Percentage of senescent cells counted at 11th passage. The number of cells expressing *β*-galactosidase is significantly higher in WJ-MSC(E) than WJ-MSC(M). The results are presented as mean values of 3 experiments ± SD, ^*∗∗∗*^ *p* < 0.001. (e) Quantification of CFU frequency. WJ-MSC isolated by mechanical method have significantly greater ability to create colonies. The results are presented as mean values of 3 experiments ± SD; ^*∗*^ *p* < 0.05; ^*∗∗*^ *p* < 0.01; ^*∗∗∗*^ *p* < 0.001.

As mentioned above the dynamics of WJ-MSC growth in both types of culture showed uneven tendency to change during the time of cultivation. Therefore, routine proliferation analysis was performed up to the latest 11th passage in both cell lines. During the first three passages, as already mentioned, enzymatically isolated WJ-MSC_E_ proliferated faster and PDT was found significantly shorter than in case of WJ-MSC_M_ population (Figure 2(a)). However, since 4th passage this tendency has been successively reversed due to slowing down of WJ-MSC_E_ proliferation related to their fastened senescent and/or increased elimination. Accordingly, WJ-MSC_E_ populations started to be dominated by the big, flattened cells with well visible cytoskeleton and other typical signs of premature cell aging. In contrast, any essential phenotypic as well as PDT changes have been noticed in parallel growing WJ-MSC_M_. Furthermore, biphasic growth rate of WST1-based analysis noticed only in the case of WJ-MSC_E_ (Figure 2(b)) may indicate lower basal cell metabolism beside of decreased number of proliferating cells as assessed by Ki67 immunoreactivity (Figure 2(c)).

Lower efficiency and fastened senescence of WJ-MSC_E_ culture have been additionally proven by increased *β*-galactosidase reactivity (Figure 2(d)) as well as lowered CFU generation potential (Figure 2(e)) observed in this cell culture. The ability of MSC population to create CFU (*colony-forming unit-fibroblasts*) is one of the most specific features of the cells in culture [7]. The assay is recommended by ISCT to define the content of stem/progenitor cells in the whole heterogeneous MSC population thus predicting expansion and longevity of the cultured cell line. Our results indicate that WJ-MSC_M_ isolated mechanically do contain significantly higher fraction of the genuine stem cells (7.5% ± 0.08 versus 5.4% ± 0.05, resp.) (Figures 2(d) and 2(e)).

#### 3.1.3. Multilineage Mesodermal and Neural Differentiation Induced by Temporal Cell Growth in Nonphysiological 21% O_2_ Conditions

**Figure 3:**
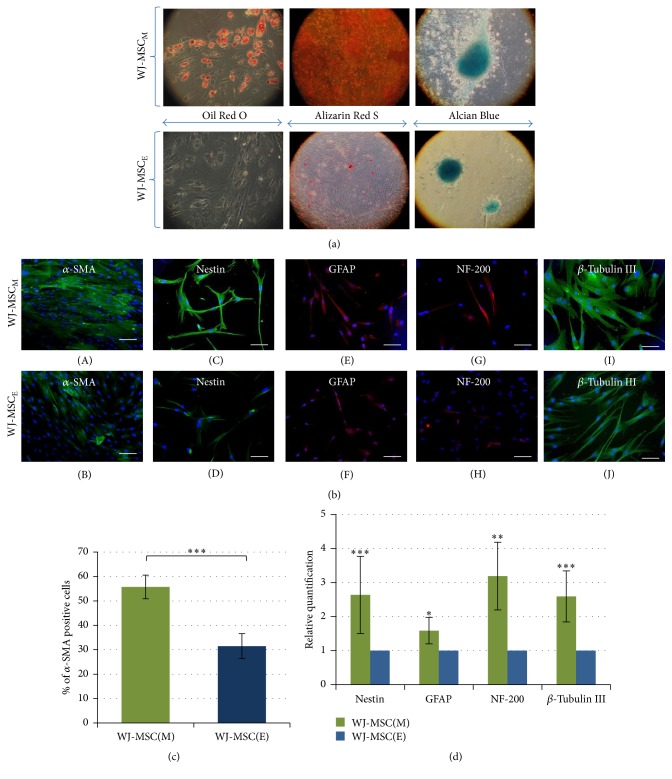
(a) Multilineage mesodermal differentiation potential of WJ-MSC. Positive results of differentiation were noticed exclusively in WJ-MSC(M) with only exception of slightly positive chondrogenesis observed in WJ-MSC(E) cultures. (b) Neural/neuronal differentiation of WJ-MSC. Expression of neural/neuronal markers was found on higher levels in mechanically than enzymatically isolated cells. Scale bars: 100 *μ*m. (c) *α*-SMA expression analysis. In mechanically isolated MSC a higher expression of primitive cell marker *α*-SMA has been observed. The results present mean values of 3 isolations ± SD, ^*∗∗∗*^ *p* < 0.001. (d) Quantitative neural/neuronal markers mRNA analysis. Relative genes expression of WJ-MSC(M) at 2nd-3rd passages normalized to the reference gene ACTB was compared to WJ-MSC(E) as calibrator group. The results are presented as mean values of 3 isolations ± SD; ^*∗*^ *p* < 0.05; ^*∗∗*^ *p* < 0.01; ^*∗∗∗*^ *p* < 0.001.

Comparison of the above two lines confirmed that only WJ-MSC_M_ have ability to differentiate toward adipocyte, osteocyte, and chondrocyte lineage* in vitro* (Figure 3(a)). Moreover, it was necessary to change the 5% O_2_ culture atmosphere employed for cell expansion to air condition during the time of mesodermal lineages differentiation.

Interestingly, similar limitation in the phenotypic plasticity of enzymatically isolated umbilical cord cell cultures was observed also in case of proneural differentiation. Again, only WJ-MSC_M_ displayed spontaneous ability to express primitive neural lineage progenitor markers like *α*-SMA (Figures 3(b) and 3(c)) and Nestin (Figures 3(b) and 3(d)) as well as neural/neuronal markers (GFAP, *β*-Tubulin III, and NF-200) (Figures 3(b) and 3(d)). Their induction was confirmed on protein and mRNA expression levels (Figures 3(b) and 3(d)). In agreement with our previous results [6] differentiation of WJ-MSC_M_ toward neural direction was observed, as in the case of mesodermal lineage differentiation, only in 21% O_2_ culture.

### 3.2. Effect of Different Oxygen Environment on the Quality and Safety of MSC Culture

**Figure 4:**
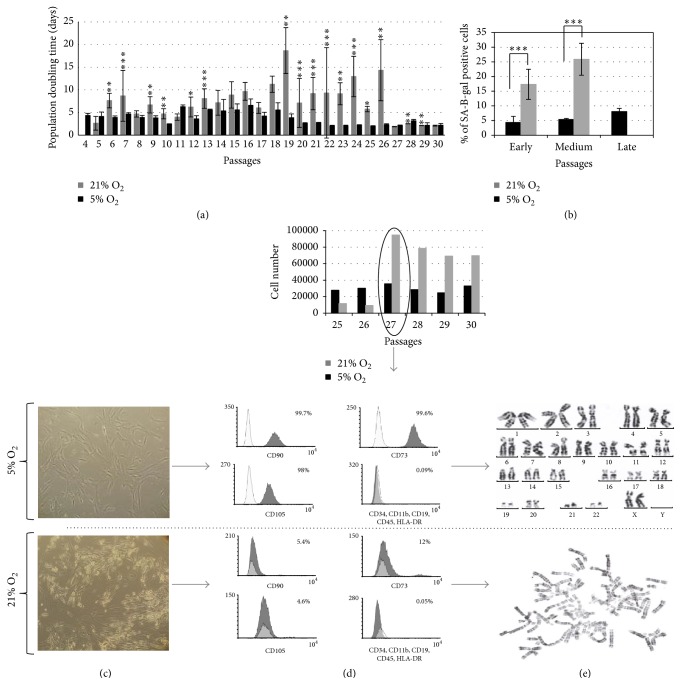
(a) The influence of physioxic 5% O_2_ or normoxic 21% O_2_ atmosphere on the quality of mesenchymal stem cell populations. AD-MSC cultured in 5% O_2_ retain relatively stable proliferation rate, when AD-MSC culture in 21% O_2_ atmosphere retain unstable, oscillating growth rate with the estimated population doubling time apparently falling down and rising again successively. (b) Percentage of senescent cells. In 21% O_2_ atmosphere the increased number of SA-*β*gal expressing cells was observed relative to cells cultured in 5% O_2_ atmosphere. (c) The morphology of AD-MSC in physioxic and normoxic conditions. Whereas in the physioxic conditions AD-MSC retained spindle-shaped morphology during the whole estimated period, in normoxic conditions they firstly acquired typical feature of aging cells and then their phenotype changed. (d) Flow cytometry analysis. After long-term culture in 21% O_2_ atmosphere we have noticed disturbed expression of MSC-specific surface antigen pattern, including complete lack of CD90, CD105, and CD73. AD-MSC growing in 5% O_2_ by the same number of passages still presented correct CD90, CD105, and CD73 antigens pattern with typical lack of hematopoietic markers, CD34, CD11B, CD19, CD45, and HLA-DR expression. (e) The comparative chromosome analysis of AD-MSC cultures growing 28 passages in either 5% O_2_ or 21% oxygen atmospheres. The cells growing in the physioxic conditions demonstrated proper karyotypes, whereas AD-MSC cultured in the air environment displayed severe karyotyping abnormalities, such as polyploidy of chromosomes (20 in 30 analyzed metaphases) and haploid chromosome numbers (10 in 30 analyzed metaphases); ^*∗*^ *p* < 0.05; ^*∗∗*^ *p* < 0.01; ^*∗∗∗*^ *p* < 0.001.

In previous paragraph we have described stimulatory effect of low oxygen condition on the growth rate in both types of differently isolated WJ-MSC cultures (Figure 1(a)). This aspect was further falsified using long-lasting AD-MSC culture. The influence of physioxic 5% O_2_ or air 21% O_2_ conditions on the quality of mesenchymal stem cell populations in respect of their stemness-related feature, multilineage differentiation ability, rate of cell senescence, and time-related karyotype stability was evaluated (Figure 4).

We found that AD-MSC cultured in 5% O2 retain, with exception of the most early passages, relatively stable proliferation rate with PDT duration around 2 days (Figure 4(a)). The parallel AD-MSC culture in 21% O_2_ atmosphere displayed much more unstable, oscillating growth rate with the estimated population doubling time apparently falling down and rising again successively. Concomitantly, the morphology of this cell population started to change significantly. Whereas in cultures at 5% O_2_ the AD-MSC maintained well their typical, spindle-shaped morphology during the whole period of measurement (Figure 4(c)), parallel growing 21% O_2_ cultures firstly acquired typical feature of aging with increasing number of SA-*β*gal positive cells (Figure 4(b)) and then displayed several incidences of temporal growth disturbances (Figure 4(a)). At 27th passage this unstable growth of cells ended with the final rapid acceleration of proliferation with PDT value failing from 14 days down to 2 days, respectively. It was followed by phenotypic changes of the most probably transformed cells (Figure 4(c)). They acquired comparatively smaller and predominantly round-shaped body floating frequently in the growth media. Concomitantly we have found heavily disturbed expression of MSC-specific surface antigen pattern, including complete lack of the main markers: CD90, CD105, and CD73 immunoreaction. Parallel AD-MSC cultures grown in 5% O_2_ atmosphere by the same number of passages still presented correct CD90, CD105, and CD73 antigens pattern with typical lack of hematopoietic markers for MSC culture, CD34, CD11B, CD19, CD45, and HLA-DR expression (Figure 4(d)).

The comparative chromosome analysis of AD-MSC cultures growing by 28th passages in either 5% O_2_ or 21% oxygen atmospheres demonstrated the nice preservation of proper karyotypes (result of testing of 5 metaphases/culture) in the physioxic culture conditions. In contrast, the population of AD-MSC cultured in the air environment displayed severe karyotyping abnormalities, such as polyploidy of chromosomes (20 in 30 analyzed metaphases) and haploid chromosome numbers (10 in 30 analyzed metaphases) (Figure 4(e)).

## 4. Discussion

### 4.1. Critical View on the Selection Criteria of Optimal MSC Derivation Method

Apart from the results of the flow cytometry analysis confirming minimal criteria for the MSC specification by typical marker expression pattern established by ISCT (2006) and capability of mesodermal lineage differentiation, the other most acknowledged proper determining positive selection of particular methodology is cell culture proliferative efficiency [8]. The high number of cell derivations is critically needed due to limited access to human tissue-based cell sources and clinical demands of rather high cell doses determining effective therapy.

However, because of enormous heterogeneity of MSC-like populations isolated from different tissues, lack of sharp definitions, and close similarity between MSC and fibroblastic primary culture in respect of their morphological and even functional characteristics [9, 10] the therapeutically desired MSC characteristic must be each time carefully verified. It should be done on the grounds of the true stemness-linked properties attributed selectively for MSC but never for cultures with dominating fibroblast feature. On the basis of our long-lasting experience with WJ-MSC culture [3, 11, 12] we though that comparison of two routinely used methods of these cells derivation presented here may illustrate well the problem of the proper MSC phenotype identification (Figure 1). These results may reassert again the reader that multipotent stem cell characteristic must be verified each time not only by flow cytometric data but also by classical test of cell differentiation toward three basic mesodermal lineages, leading to formation of osteogenic, adipogenic, or chondrogenic cell phenotypes. Additionally, the specificity of WJ-MSC ability to acquire neural-like phenotypes (Figures 3(b), 3(c), and 3(d)) [3, 6] when air condition is used for differentiation can be observed only in case of mechanically derived WJ-MSC_M_. The lack of capability of WJ-MSC_E_ culture of multilineage differentiation together with drastic decrease of cells CFU formation efficiency (Figures 2(d) and 3(a)) leads us to conclusion that, despite of initially higher proliferation rates observed at first passages as well as correct phenotype pattern in immunocytochemical cell staining and cytometric screening (Figures 1(b) and 1(c)), this culture does not fulfill adequately criteria needed for its qualification as therapeutic MSC. It would rather indicate predominance of the fibroblasts present in all stromal tissues. The further passaging of both types of differently isolated WJ-MSC cultures confirmed again that only the first mechanical method can provide steadily proliferating cells for longer time period. Also the number of viable cells determined by enzyme-linked assay with WST-1 reagent showed significantly higher ratio of living cells in WJ-MSC_M_ culture comparing to WJ-MSC_E_. Interestingly this was not correlated well with the cell cycle activity determined by Ki67 expression (Figure 2(c)) suggesting either increased cells death by apoptosis or eventually earlier contact inhibition in this type of culture.

Concomitantly with the above observations the senescence-associated *β* galactosidase (SA-*β*gal) positive cells number was significantly higher in the enzymatically isolated cell populations than in WJ-MSC_M_ cultures (Figure 3(e)). In parallel the WJ-MSC_E_ growth rate decreased steadily with PDT value increasing from 1.5 days on the beginning to over 3 days at the last estimated passages (Figure 2(a)). This correlates well with increased signs of cell senescence and lowered CFU formation efficiency implying lower capability of WJ-MSC_E_ of stem cells specific self-renewal.

### 4.2. Influence of Oxygen Atmosphere on Proliferation and Genetic Stability* In Vitro*

Oxygen concentration is one of the most important determinants of tissue metabolism precisely regulated in all organisms. Within the various tissues stem cells niches in mammals oxygen is present in strictly controlled concentration being “hypoxic” in relation to atmospheric air but “physioxic” according to* in situ* MSC demands [13–15]. Thus, cultures growing in air environment (21%  O_2_), still being routine in majority of laboratories, must be considered as nonphysiological and inducing oxidative stress in cultured cells with increased production of reactive oxygen species (ROS) with destructive impact on the majority of intracellular lipids, proteins, and DNA macromolecules. Consequently, after final failing of antioxidative defense systems, the cells would induce specific programs leading directly to senescent and apoptotic death [16].

To characterize and compare influence of either 5% or 21% oxygen microenvironment in our experiments with MSC long-term cultures we have used AD-MSC isolated by the commercial method of AD-MSC isolation (CellCelution Cytori®) routinely employed in various clinical therapeutic purposes [17, 18]. After running the cultures up to 30 passages under both oxygen concentrations we have found all estimated parameters stable and unchanged but exclusively in the case of 5% oxygen cultures. These cells displayed proper pattern of all specific MSC markers expression (Figure 4(d)), unchanged plasticity for differentiation into the three germ layers (not shown), and stable, correct karyotype (Figure 4(e)).

Serious safety problems have appeared exclusively in the cultures growing under 21% oxygen concentration. This observation is in agreement with a concept of deteriorating influence of oxidative stress induced by the culture environment being far from physiological oxygen concentration [19–22]. The abrupt acceleration of growth parameters encountered here between 26 and 27 passages (Figure 4(c)) was preceded by several waves of transient proliferation slowness probably due to successive mitotic crises (Figure 4(a)). The cultures displaying similar instability of growth rate during long-term passaging would deserve special attention and additional safety control from the laboratory staff. Usually such type of behavior has been linked with accumulation of genetic mutations leading to aneuploidy and heavy karyotype destruction (Figure 4(e)) suggesting possibility of the spontaneous oncogenic cell transformation. Therefore such cultures, beside even excellent further proliferation, should be excluded from any practical use.

Here we should mention that during preclinical experiments we are often concerned with uncontrolled and expansive cell growth being occasionally observed after intraparenchymal transplantation of multi- or pluripotential stem cell populations as described in classical work of Erdö et al. [23] or restricted, spontaneously transformed progenitors OPC described in Hansmann et al. work [24]* in vivo* and by Ben-David et al. [25] in somatic progenitors* in vitro*. Such cells after transplantation either can form histologically determined tumors or in contrast might expand locally on expense of surrounding host tissue under influence of not fully determined intrinsic or environmental cues [26–28]. This reaction has been observed in various experimental mammalian models and depends on secretion of stimulatory growth or trophic factors playing inductive function either during physiologic proliferative peak under brain development* in vivo* [29] or in stem cell cultures expanding* in vitro*. However, it is still unknown and highly controversial when and under what circumstances these proliferative reactions can cross the border between strictly controlled, physiological responses to stimuli and oncogenic transformation [28]. From this point of view it would be important to determine if somatic MSCs growing long time in unfavorable, reactive oxygen producing air atmosphere may spontaneously transform toward tumorigenic direction as suggested by our (Figures 4(c), 4(d), and 4(e)) and other results describing spontaneous transformation of human mesenchymal MSC and neural NSC as well as oligodendrocyte progenitor OPCs in long-term cultures [24, 30, 31]. However there are still contradictory reports concerning the same issue [32]. The answer to this important question in our case would need more hard arguments from the further molecular [24, 27], genetic [33], and routine animal-based tumorigenicity testing.

Summarizing, we have shown here that long time cell expansion in not optimally chosen culture condition can seriously hamper numerous important properties of MSC cultures. Thus, practically thinking, any type of laboratory derived cells must be each time very carefully verified by adequately chosen and accepted control methods. On the other hand culturing of cells in lowered (5%) oxygen atmosphere should desire more general acceptance and propagation as a relative simple and efficient method for improving various critical growth parameters including cell karyotype stabilization.

It is also worth to notice that although enzymatic WJ-MSC isolation gives comparatively better initial efficiency, only mechanically isolated WJ-MSC ensures long-lasting cell proliferation capacity, higher differentiation potential, and evidently slower cell senescence. Thus, we would like again to highlight the urgent necessity to implement more sensitive and selective methods for prediction and control the fate of therapeutic cell* in vitro* before their use in human clinic.

## Figures and Tables

**Table 1:** Primary antibodies used for immunocytochemistry.

Primary antibody	Source	Isotype	Dilution	Company
CD73	Mouse monoclonal	IgG3	1 : 200	Santa Cruz
CD90	Mouse monoclonal	IgG1	1 : 200	Santa Cruz
Collagen	Goat polyclonal	IgG (H + L)	1 : 200	Santa Cruz
Vimentin	Mouse monoclonal	IgG1	1 : 100	Dako
Fibronectin	Rabbit polyclonal	IgG (H + L)	1 : 200	Dako
Ki67	Mouse monoclonal	IgG1	1 : 400	Novocastra
*α*-SMA	Mouse monoclonal	IgG2a	1 : 200	Sigma
Nestin	Mouse monoclonal	IgG1	1 : 200	Millipore
GFAP	Rabbit polyclonal	IgG (H + L)	1 : 500	Dako
NF-200	Mouse monoclonal	IgG1	1 : 400	Sigma
*β*-Tubulin III	Mouse monoclonal	IgG2b	1 : 1000	Sigma

**Table 2:** Primers used for quantitative RT-PCR.

Gene	Product size	Primer sequence (5′ → 3′)
CD73	241 bp	F: CGCAACAATGGCACAATTAC
		R: CTCGACACTTGGTGCAAAGA

CD90	236 bp	F: CTAGTGGACCAGAGCCTTCG
		R: TGGAGTGCACACGTGTAGGT

CD105	165 bp	F: CACTAGCCAGGTCTCGAAGG
		R: CTGAGGACCAGAAGCACCTC

CD166	217 bp	F: CGCAATGCAACAGGAGACTA
		R: GGCTAGATCGAAGCCTGATG

Vimentin	170 bp	F: GAGAACTTTGCCGTTGAAGC
		R: TCCAGCAGCTTCCTGTAGGT

Collagen	332 bp	F: AGTGGTTACTACTGGATTGACC
		R: TTGCCAGTCTCCTCATCC

Fibronectin	386 bp	F: CTGGGATGCTCCTGCTGT
		R: CTGTTTGATCTGGACCTGCAG

Nestin	169 bp	F: TGGCTCAGAGGAAGAGTCTGA
		R: TCCCCCATTTACATGCTGTGA

GFAP	266 bp	F: GCAGAGATGATGGAGCTCAATGACC
		R: GTTTCATCCTGGAGCTTCTGCCTCA

NF-200	160 bp	F: GAGGAACACCAAGTGGGAGA
		R: TTCTGGAAGCGAGAAAGGAA

*β*-Tubulin III	159 bp	F: CTCAGGGGCCTTGGACATC
		R: CAGGCAGTCGCAGTTTTCAC

*β*-actin (ACTB)	120 bp	F: GCCAACCGCGAGAAGATGA
		R: CATCACGATGCCAGTGGTA
